# Sustainable sport nutrition behaviors, mental resilience, and perceived athletic performance in competitive athletes

**DOI:** 10.3389/fnut.2026.1935463

**Published:** 2026-10-06

**Authors:** Fan Xitong, Miao Cheng

**Affiliations:** 1 Education and Sports School of Soochow University, Suzhou, Jiangsu, China; 2 Public Physical Education Department, Soochow College, Soochow University, Suzhou, China

**Keywords:** athletes, mental resilience, mixed methods, perceived performance, recovery quality, sport psychology, sustainable sport nutrition

## Abstract

**Background:**

Sustainable dietary practices may influence athlete health, recovery, psychological stability, and environmental responsibility; however, evidence regarding their relationship with mental resilience and perceived athletic performance remains limited.

**Objective:**

This study investigated associations among sustainable sport nutrition practices, sustainability awareness, recovery quality, mental resilience, and perceived athletic performance in competitive athletes.

**Methods:**

A cross-sectional mixed-methods study was conducted over 8 weeks. Of 300 invited athletes, 230 agreed to participate and 202 completed the full survey. Participants completed self-report measures assessing sustainable sport nutrition practices, sustainability awareness, recovery quality, mental resilience, and perceived athletic performance. Semi-structured interviews were conducted with 30 survey completers. Quantitative data were analyzed using descriptive statistics, Pearson correlations, and multiple linear regression, while qualitative data were examined using reflexive thematic analysis and integrated through a joint display.

**Results:**

Sustainable sport nutrition practices were positively associated with mental resilience (*r* = 0.47, *p* < 0.001), recovery quality (*r* = 0.43, *p* < 0.001), and perceived athletic performance (*r* = 0.39, *p* < 0.001). Sustainability awareness was associated with sustainable nutrition practices (*r* = 0.51, *p* < 0.001). Regression analysis showed that sustainable sport nutrition practices (β = 0.32, *p* < 0.001), recovery quality (β = 0.29, *p* < 0.001), and sustainability awareness (β = 0.21, *p* = 0.003) were positively associated with mental resilience, which was associated with perceived athletic performance (β = 0.36, *p* < 0.001). Qualitative findings indicated that structured nutrition routines supported mental preparation, recovery confidence, emotional regulation, and training discipline.

**Conclusion:**

Sustainable sport nutrition behaviors were associated with improved recovery quality, mental resilience, and perceived athletic performance. Findings should be interpreted as associations due to the cross-sectional design and self-report measures. Sustainable sport nutrition should emphasize performance compatibility, adequate energy availability, and sufficient protein intake rather than restrictive approaches.

## 1 Introduction

Sustainable diets have become an important topic in sports nutrition because they connect athlete health, long-term performance, and environmental responsibility. In modern sport, athletes are expected not only to perform at a high level but also to maintain physical and psychological wellbeing across repeated training and competition cycles (1). Dietary routines are central to this process because food intake affects energy availability, glycogen restoration, protein synthesis, recovery perception, mood, sleep quality, immune function, and training consistency. For this reason, sustainable nutrition in sport should not be viewed only as an environmental concept but also as a potential lifestyle behavior associated with preparation, recovery, and psychological readiness (2).

In competitive athletes, sustainable nutrition must be interpreted differently from general population dietary advice. A sustainable athlete diet should not be understood as restrictive eating, under-fueling, or simple reduction of animal protein. Rather, it should be performance-compatible: it should support energy availability, carbohydrate needs, protein quantity and quality, essential amino acid intake, hydration, micronutrient adequacy, recovery, and training adaptation while also encouraging environmentally responsible and minimally wasteful food choices (3). Plant-forward eating may be compatible with athletic performance when meals are carefully planned, energy-dense, culturally acceptable, and protein-adequate. However, poorly planned sustainable or plant-based diets may increase the risk of inadequate energy intake, insufficient high-quality protein, or low energy availability, particularly in athletes with high training loads (4).

Mental resilience is one of the most important psychological qualities in athletic performance. It refers to the ability to remain focused, confident, emotionally stable, and persistent under pressure. Resilient athletes can better manage fatigue, competition anxiety, setbacks, and training stress (5). Although mental resilience is often studied through psychological skills training, coaching environments, motivation, and coping resources, nutrition may also be related to resilience by supporting stable energy availability, recovery confidence, and perceived preparedness. Sustainable sport nutrition behavior may be associated with mental resilience through several complementary mechanisms (6). First, it may reflect self-regulation. Athletes who consistently plan meals, maintain hydration, reduce reliance on ultra-processed foods, and make deliberate food choices may also develop routines that support discipline, emotional control, and training consistency (7).

Second, sustainable food choices may involve value-based alignment. When athletes perceive their dietary behavior as beneficial for personal health, performance longevity, and environmental responsibility, food choices may become connected with identity, purpose, and intrinsic motivation. Third, sustainable diets may relate to biological and recovery-related pathways (8). Nutrient-dense meals, adequate carbohydrate and protein intake, regular hydration, and lower reliance on highly processed foods may help stabilize energy levels, support sleep and recovery perception, and influence mood-related processes, including possible gut-brain interactions. These mechanisms provide a theoretical basis for examining why sustainable sport nutrition practices may be associated with mental resilience while recognizing that cross-sectional data cannot confirm causal direction. Previous studies have examined sport nutrition knowledge, dietary habits, hydration, supplementation, motivation, and performance outcomes (9). However, the relationship between sustainable sport nutrition behaviors and mental resilience remains underexplored. Many athletes understand the importance of healthy eating, but they may not connect sustainable food choices with psychological outcomes such as discipline, confidence, stress regulation, and performance readiness. This gap is important because sustainable sport nutrition may contribute to a more holistic model of athletic performance, combining physical recovery, mental stability, and value-based motivation (10).

### 1.1 Novelty and contribution of the study

The novelty of this study lies in its integration of sustainable sport nutrition, mental resilience, recovery quality, and perceived athletic performance within a single athlete-centered framework. Existing sport nutrition research has mainly emphasized nutrient intake, supplementation, hydration, and body composition, whereas the present study examines sustainable sport nutrition as both a behavioral and psychological construct. This study makes three specific contributions. First, it examines sustainable sport nutrition as a practical behavior that must remain compatible with athlete energy and protein demands. Second, it evaluates whether recovery quality and mental resilience are statistically associated with the relationship between sustainable sport nutrition practices and perceived performance in a cross-sectional model. Third, it combines quantitative analysis with qualitative interviews, allowing the study to connect statistical associations with athlete experiences of self-regulation, recovery confidence, emotional control, and value-based motivation.

## 2 Materials and methods

### 2.1 Study design and duration

This study used a cross-sectional mixed-methods design. The quantitative component involved a structured questionnaire, while the qualitative component included semi-structured interviews. The study was conducted over 8 weeks. A pragmatic mixed-methods approach was selected because sustainable sport nutrition behavior, recovery quality, mental resilience, and perceived athletic performance are measurable constructs, while athlete perceptions require qualitative explanation (11). Because the study was cross-sectional, all statistical models were interpreted as associations rather than causal, temporal, or mediation pathways.

### 2.2 Participants, setting, and sample size

Participants were university, club/regional, and national-level competitive athletes recruited from university teams, sports clubs, and training centers. The sample should be interpreted as a non-professional competitive athlete cohort, with most participants competing at university or club/regional level and a smaller proportion competing at national level. A total of 300 athletes were invited to participate. Of these, 230 athletes agreed, and 202 completed the full questionnaire. The final analytical sample represented 67.3% of all invited athletes (202/300) and 87.8% of athletes who agreed to participate (202/230). The final sample included 108 male athletes (53.5%) and 94 female athletes (46.5%), with a mean age of 21.4 ± 2.6 years. Participants had an average training experience of 5.8 ± 2.4 years and trained 4.7 ± 1.3 sessions per week, with an average weekly training duration of 9.2 ± 3.8 h. For the qualitative component, 30 athletes were selected for interviews, representing 14.9% of the final survey sample.

An *a priori* sample size estimation was considered for the regression-based analysis. Assuming a medium effect size, α = 0.05, statistical power = 0.80, and five predictors, the required minimum sample was estimated to be approximately 150–170 participants. Therefore, the final sample of 202 athletes was considered adequate for the primary correlation and regression analyses. For the qualitative component, 30 athletes were selected from the completed survey group using purposive sampling. Selection aimed to include variation in gender, sport type, competition level, and sustainable sport nutrition practice scores so that the interviews could capture a range of nutrition experiences rather than only the views of athletes with high sustainable nutrition scores.

### 2.3 Inclusion and exclusion criteria

Athletes were included if they were aged 18 years or above, had at least 1 year of organized training experience, trained at least 3 times per week, and provided informed consent. Athletes were excluded if they reported a severe injury, a medically prescribed restrictive diet, a chronic illness affecting food intake, or incomplete questionnaire responses.

### 2.4 Data collection procedure

Participants completed either an online or paper-based questionnaire. The questionnaire required approximately 15–20 min to complete. After the survey, 30 athletes were invited to participate in semi-structured interviews lasting 20–30 min. Interview questions explored usual dietary patterns, sustainable food choices, perceived effects of diet on mood and focus, recovery confidence, competition readiness, and barriers to sustainable eating. All responses were anonymized before analysis.

### 2.5 Measurement instruments

Sustainable dietary practices were measured using a study-specific Sustainable Sport Nutrition Practice Score developed for the present athlete sample. The score was designed to capture sustainable and performance-supportive nutrition behaviors rather than restrictive dietary behavior. Because sustainable sport nutrition is a multidimensional concept involving both dietary sustainability and performance-supportive behaviors, this exploratory index was developed to capture practical nutrition behaviors relevant to athletes rather than to represent a fully validated measure of environmental sustainability. Therefore, the included items should be interpreted as an initial behavioral framework requiring further psychometric evaluation. Items assessed plant-forward meals, whole or minimally processed food choices, reduced ultra-processed food intake, balanced meal planning, protein adequacy from diverse sources, local or seasonal food choices, food-waste reduction, and hydration planning. The scale used a 5-point Likert response format from 1 = never to 5 = always. Total scores ranged from 8 to 40, with higher scores indicating more frequent sustainable sport nutrition practices.

For visual comparison with other 1–5 scale variables, the Sustainable Sport Nutrition Practice Score was converted to a 1–5 mean item score using the formula: standardized score = total Sustainable Sport Nutrition Practice Score/8. Thus, the mean total score of 27.6 out of 40 was displayed as 3.45 on the 1–5 scale in Figure 1. Hydration planning was included because consistent fluid intake is an important sport nutrition behavior supporting recovery and training readiness. However, this item does not directly represent ecological sustainability indicators, such as reusable bottle practices, water conservation, or reduction of single-use plastic consumption. Therefore, hydration was considered a performance-supportive nutrition behavior within the exploratory index rather than a direct measure of environmental sustainability. Future versions of the instrument should separate sport hydration behaviors from ecological sustainability dimensions.

**Figure 1:**
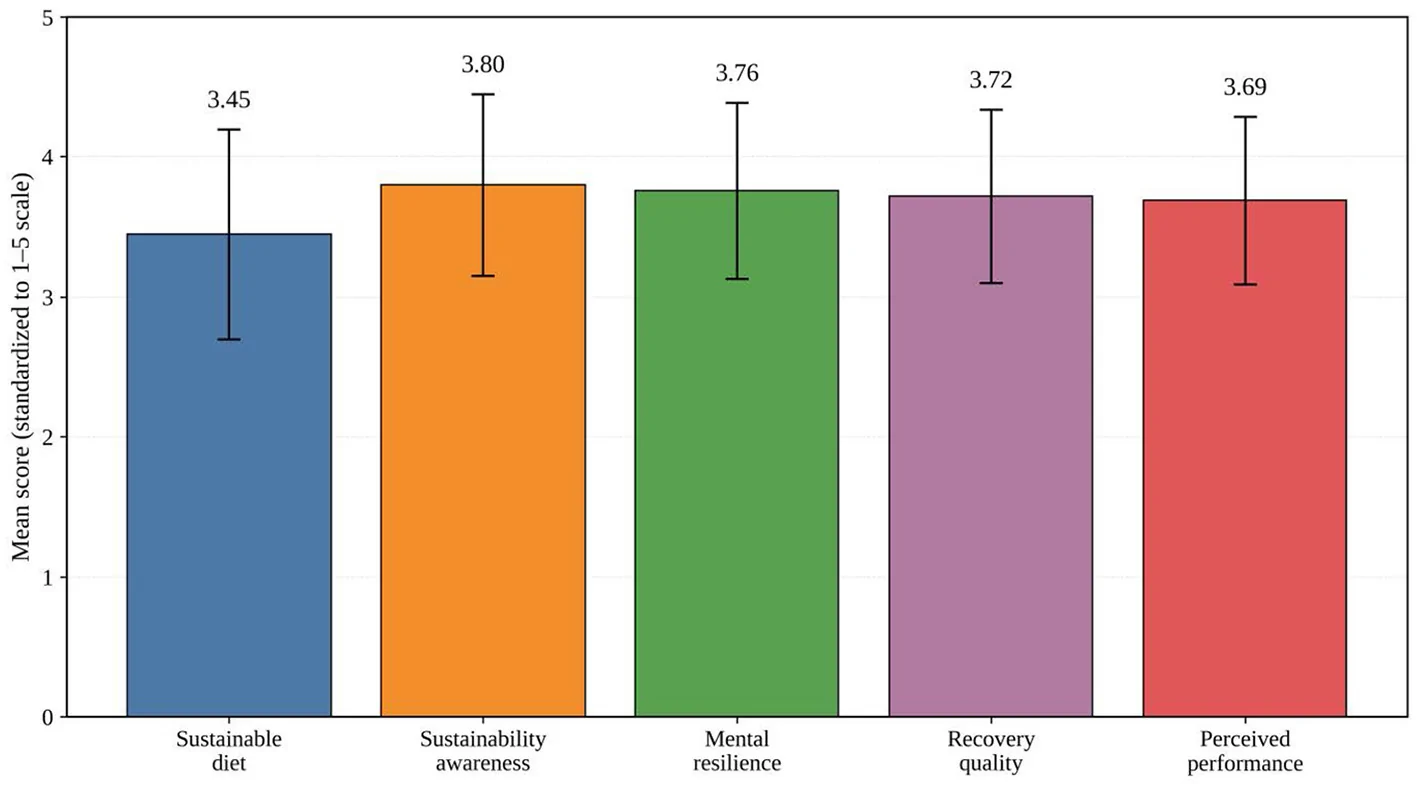
Mean scores of sustainable sport nutrition practices, sustainability awareness, mental resilience, recovery quality, and perceived athletic performance.

The Sustainable Sport Nutrition Practice Score was treated as a study-specific behavioral index rather than a fully validated diagnostic scale. Accordingly, the Sustainable Sport Nutrition Practice Score was developed as an exploratory behavioral measure for the present study rather than as a standardized psychometric instrument. The reported Cronbach's alpha value (α = 0.84) indicates acceptable internal consistency among the eight items but does not provide evidence of construct validity, factorial structure, convergent validity, or criterion validity. It was not designed to diagnose dietary adequacy, energy availability, leucine intake, amino acid distribution, or risk of low energy availability. Internal consistency in the present sample was acceptable (Cronbach's alpha = 0.84). Mental resilience was measured using items related to emotional control, focus, confidence, stress tolerance, persistence, and recovery from setbacks. The mental resilience scale showed strong internal consistency (Cronbach's alpha = 0.88). Recovery quality was assessed through self-reported fatigue control, sleep quality, muscle soreness, and energy restoration (Cronbach's alpha = 0.80).

Perceived athletic performance was assessed through training consistency, competition readiness, perceived improvement, and confidence in performance (Cronbach's alpha = 0.82). Sustainability awareness was measured through items related to food source, environmental responsibility, food waste, and sustainable food choices. Cronbach's alpha was used only as an indicator of internal consistency, not as evidence of complete psychometric validation.

### 2.6 Statistical analysis

Data were analyzed using SPSS version 27. Descriptive statistics were calculated as mean, standard deviation, frequency, and percentage. Pearson correlation analysis was used to examine associations between sustainable sport nutrition practices, sustainability awareness, mental resilience, recovery quality, processed food intake, and perceived athletic performance. Multiple linear regression was used to identify variables statistically associated with mental resilience and perceived athletic performance. Statistical significance was set at *p* < 0.05. Two multiple linear regression association models were conducted. In Model A, mental resilience was entered as the dependent variable, and the five independent variables were sustainable sport nutrition practices, recovery quality, sustainability awareness, processed food intake, and training experience. In Model B, perceived athletic performance was entered as the dependent variable, and the five independent variables were mental resilience, recovery quality, sustainable sport nutrition practices, processed food intake, and training experience. These variables were selected based on the study framework and the observed correlation pattern. Multicollinearity should be assessed using tolerance and variance inflation factor (VIF) values. Tolerance values above 0.20 and VIF values below 5.00 were considered acceptable.

Exploratory factor analysis (EFA) was conducted to examine the underlying dimensional structure of the Sustainable Sport Nutrition Practice Score. Sampling adequacy was evaluated using the Kaiser–Meyer–Olkin (KMO) measure and Bartlett's test of sphericity. Factors were extracted using principal axis factoring with an appropriate rotation method, and items with factor loadings below the predefined threshold were examined for potential removal.

### 2.7 Qualitative analysis

Qualitative interview data were analyzed using reflexive thematic analysis (12). A pragmatic and critical realist orientation was adopted, meaning that athlete narratives were interpreted as meaningful accounts of lived sport nutrition experiences while recognizing that these experiences were shaped by training demands, food environments, personal values, and performance expectations (3). Interview responses were read repeatedly to achieve familiarization. Initial codes were generated inductively from athlete responses related to dietary routines, sustainable food choices, recovery perceptions, mood, focus, performance readiness, and barriers to implementation. Codes were compared across participants and grouped into candidate themes (1, 13). Themes were reviewed against the original interview responses and refined to ensure that they captured recurring patterns and meaningful differences in athlete experience (14).

### 2.8 Ethical considerations

The study was conducted in accordance with the ethical principles and institutional requirements for research involving human participants. Ethical approval was obtained from the Research Ethics Committee of Soochow University (Approval No. SC-SU-REC-2026-0316; approved February 25, 2026). All participants provided written or electronic informed consent before participation. The study was conducted in accordance with institutional ethical requirements and the principles of voluntary participation, confidentiality, privacy protection, and the right to withdraw.

## 3 Results

### 3.1 Participant recruitment and final analytical sample

The participant recruitment process is shown in Figure 2. A total of 300 competitive athletes were invited to participate. Among them, 230 athletes agreed to participate, and 202 athletes completed the full questionnaire. The final analytical sample represented 67.3% of all invited athletes and 87.8% of athletes who agreed to participate. From the completed survey group, 30 athletes were selected for semi-structured interviews, representing 14.9% of the final analytical sample. The final sample included both male and female athletes from team and individual sports (15). The mean age of the participants was 21.4 ± 2.6 years, and the average training experience was 5.8 ± 2.4 years. Participants trained approximately 4.7 ± 1.3 sessions per week, with an average weekly training duration of 9.2 ± 3.8 h (16).

**Figure 2:**
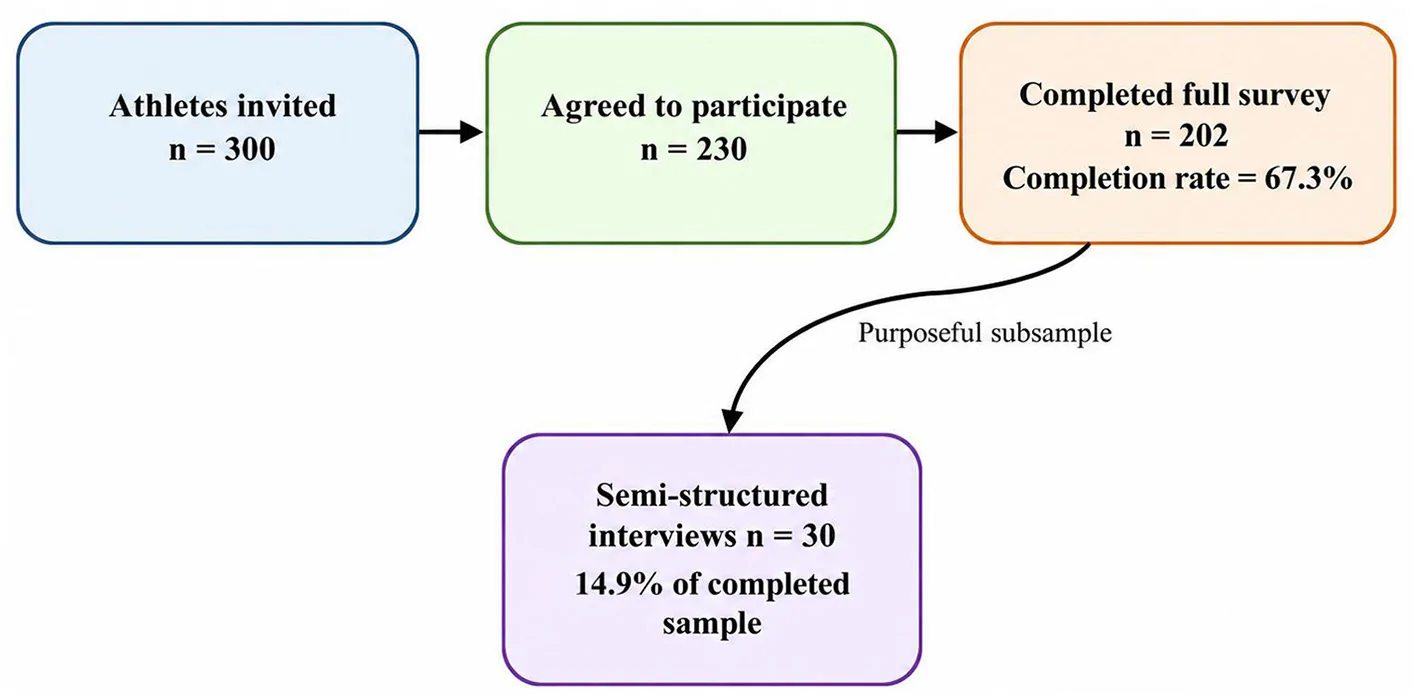
Participant recruitment flow and final analytical sample. The completion rate was 67.3% of invited athletes and 87.8% of athletes who agreed to participate.

### 3.2 Conceptual association framework

The conceptual association framework is presented in Figure 2. The framework illustrates the variables included in two independent cross-sectional regression association models. Model A examined factors statistically associated with mental resilience, whereas Model B examined factors statistically associated with perceived athletic performance. These models represent statistical associations and do not imply causal pathways, mediation, or temporal relationships as shown in Figure 3. Sustainable sport nutrition practices included plant-forward meals, regular hydration planning, reduced intake of ultra-processed foods, local or seasonal food choices, protein adequacy, meal planning, and food waste reduction. Mental resilience included focus, confidence, emotional regulation, stress tolerance, and persistence during training or competition (17). These findings indicate that sustainable sport nutrition behavior was statistically associated with perceived performance, recovery quality, and psychological preparedness; however, causal direction cannot be inferred from the cross-sectional data.

**Figure 3:**
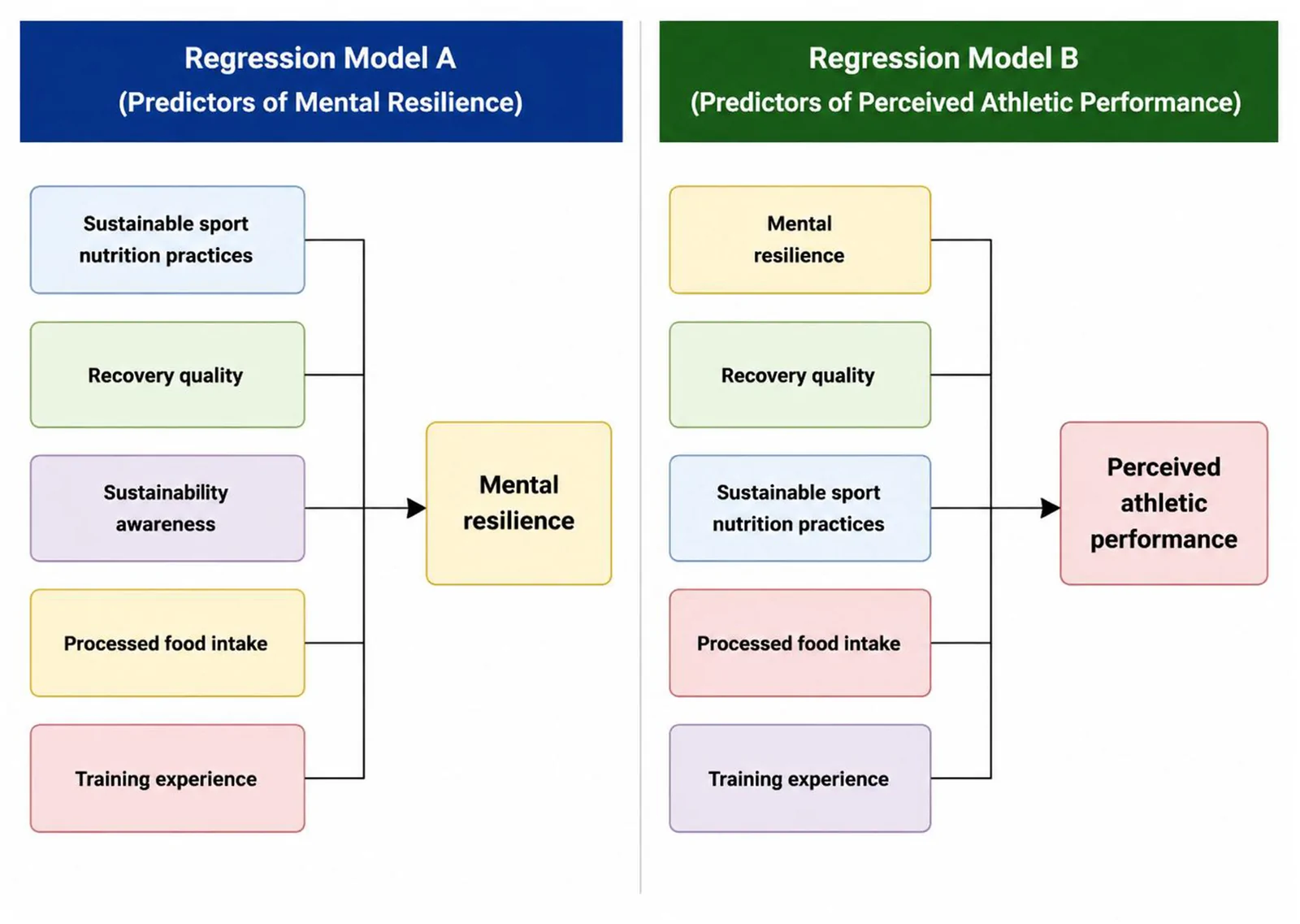
Conceptual framework illustrating the variables included in the two independent regression association models. Model A examined predictors of mental resilience, and Model B examined predictors of perceived athletic performance. The framework represents statistical associations rather than causal or mediation pathways.

### 3.3 Distribution of participants by sport type

The sport-type distribution of participants is presented in Figure 4. Football athletes represented the largest group (23%), followed by basketball (18%), athletics (16%), swimming (13%), martial arts (11%), and other sports (19%) (18). This distribution indicates that the sample included athletes from a range of sport contexts, allowing the study to examine sustainable sport nutrition behavior across endurance, skill-based, strength-related, and team-based athletic environments. The inclusion of multiple sport types is important because dietary patterns, recovery demands, and psychological stressors may differ across sports (19). Athletes in team sports may be more influenced by peer eating habits and team food environments, whereas athletes in individual sports may show greater personal control over dietary routines.

**Figure 4:**
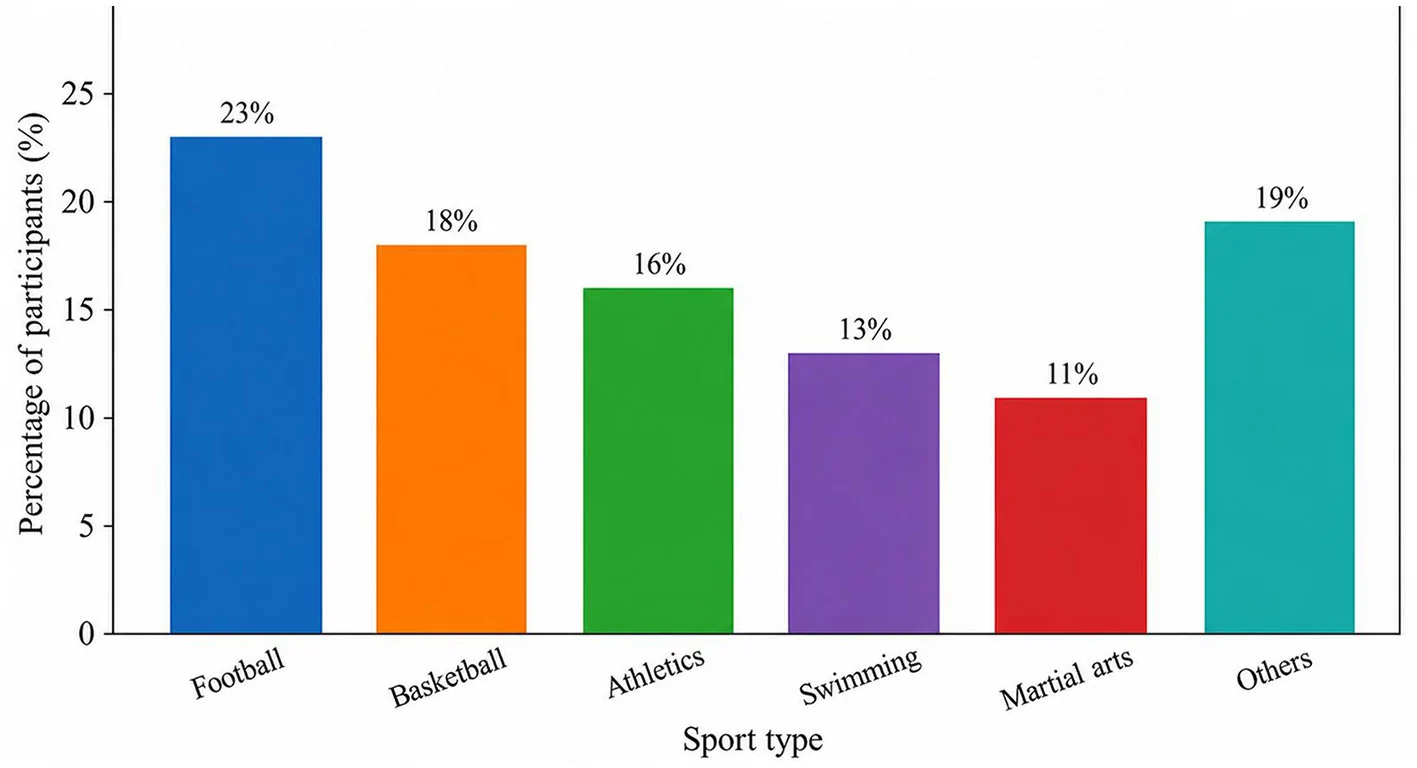
Distribution of participants by sport type.

### 3.4 Descriptive results of main study variables

The mean scores of the main study variables are shown in Figure 1. The mean Sustainable Sport Nutrition Practice Score was 27.6 ± 5.9 out of 40, which was displayed as 3.45 on the 1–5 mean item scale. Sustainability awareness had a mean score of 3.88 ± 0.66, mental resilience was 3.76 ± 0.62, recovery quality was 3.72 ± 0.61, and perceived athletic performance was 3.69 ± 0.58. These findings suggest that athletes generally showed positive awareness of sustainable sport nutrition behavior, while the actual practice level remained moderate. This indicates a potential awareness–behavior gap, where athletes may understand the importance of sustainable nutrition but may not consistently apply it due to practical barriers such as cost, time, access, taste preference, or uncertainty about protein adequacy (20).

### 3.5 Correlation analysis among sustainable nutrition, resilience, recovery, and performance

The correlation matrix is shown in Figure 5 and summarized in Table 1. Sustainable sport nutrition practices were positively associated with mental resilience (*r* = 0.47, *p* < 0.001), recovery quality (*r* = 0.43, *p* < 0.001), and perceived athletic performance (*r* = 0.39, *p* < 0.001). Sustainability awareness was associated with sustainable sport nutrition practices (*r* = 0.51, *p* < 0.001), indicating that athletes with greater environmental and nutrition awareness were more likely to report sustainable sport nutrition behaviors (21). Processed food intake showed a negative association with mental resilience (*r* = −0.31, *p* < 0.001). This association should be interpreted cautiously because processed food intake may also reflect practical constraints such as limited time, food access, cost, travel schedules, training load, or food security rather than diet quality alone (22). These contextual factors, including socioeconomic status, disposable food budget, and access to affordable nutritious foods, were not directly measured in the present study and may partially explain the observed association.

**Figure 5:**
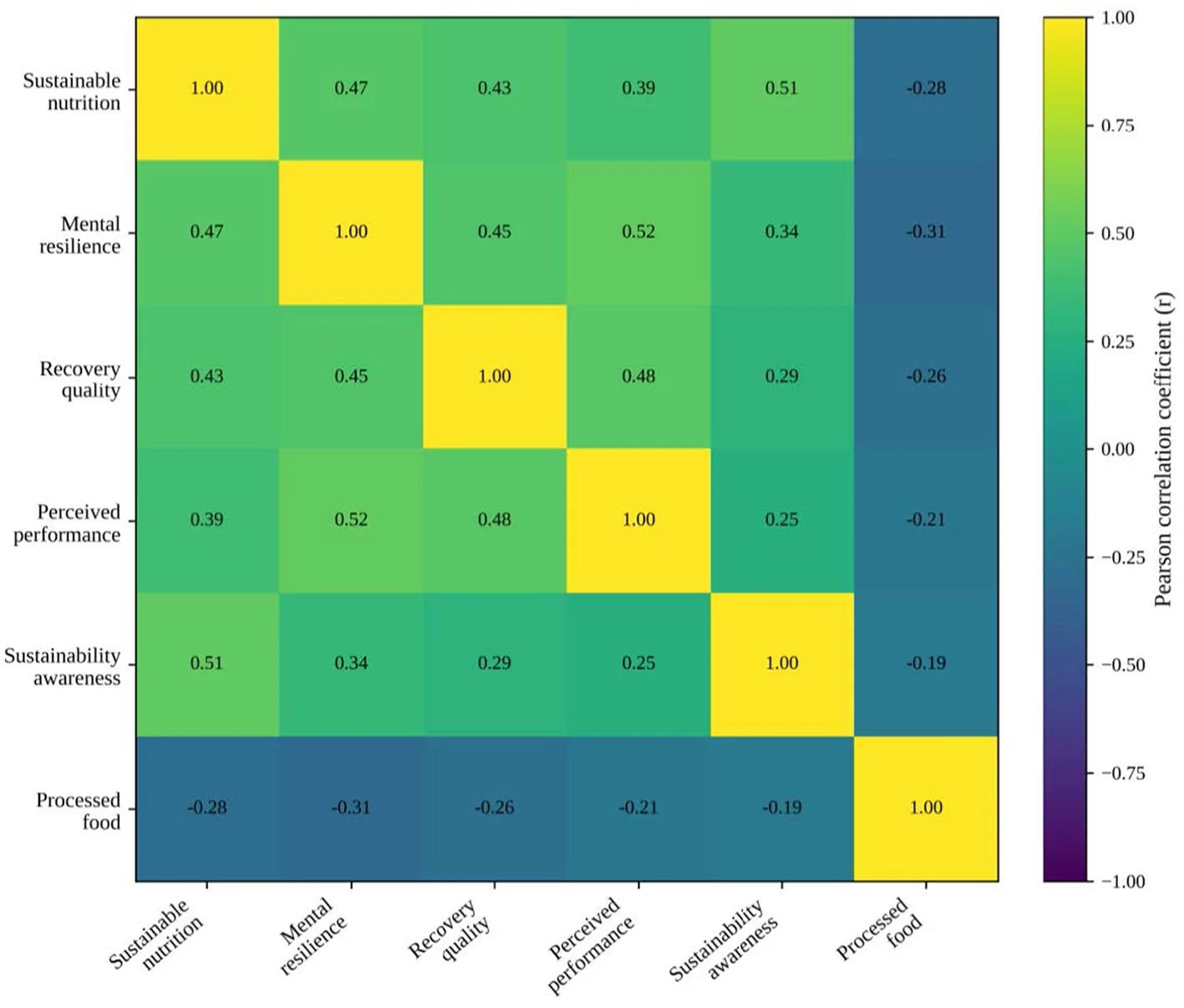
Correlation heat map among key study variables.

**Table 1:** Correlation and regression summary for sustainable sport nutrition practices, resilience, recovery, and perceived performance.

Analysis/predictor	Outcome variable	Statistical value	*p*-value	Interpretation
Sustainable sport nutrition practices	Mental resilience	*r* = 0.47	< 0.001	Moderate positive association
Sustainable sport nutrition practices	Recovery quality	*r* = 0.43	< 0.001	Moderate positive association
Sustainable sport nutrition practices	Perceived athletic performance	*r* = 0.39	< 0.001	Moderate positive association
Sustainability awareness	Sustainable sport nutrition practices	*r* = 0.51	< 0.001	Strong positive association
Processed food intake	Mental resilience	*r* = −0.31	< 0.001	Negative association
Sustainable sport nutrition practices	Mental resilience	β = 0.32	< 0.001	Positive regression association
Recovery quality	Mental resilience	β = 0.29	< 0.001	Positive regression association
Sustainability awareness	Mental resilience	β = 0.21	0.003	Positive regression association
Mental resilience	Perceived athletic performance	β = 0.36	< 0.001	Strongest regression association
Recovery quality	Perceived athletic performance	β = 0.31	< 0.001	Positive regression association
Sustainable sport nutrition practices	Perceived athletic performance	β = 0.19	0.008	Positive regression association

In addition, mental resilience showed a stronger correlation with perceived athletic performance (*r* = 0.52, *p* < 0.001) compared with the correlation between sustainable sport nutrition practices and perceived athletic performance (*r* = 0.39, *p* < 0.001), indicating that these represent distinct relationships among the study variables as shown in Table 2.

**Table 2:** Participant demographic and sport characteristics.

Variable	Category/ value	*n* (%) or Mean ±SD
Total participants	Completed survey	202
Age	Years	21.4 ± 2.6
Gender	Male	108 (53.5%)
Gender	Female	94 (46.5%)
Training experience	Years	5.8 ± 2.4
Weekly training frequency	Sessions/week	4.7 ± 1.3
Weekly training duration	Hours/week	9.2 ± 3.8
Sport level	University level	96 (47.5%)
Sport level	Club/regional level	74 (36.6%)
Sport level	National level	32 (15.8%)
Sport type	Team sports	116 (57.4%)
Sport type	Individual sports	86 (42.6%)

### 3.6 Cross-sectional regression association model

The cross-sectional regression association model is presented in Figure 6. The first regression model examined variables statistically associated with mental resilience. The model was statistically significant, *F*(5,196) = 22.84, *p* < 0.001, and accounted for 37% of the variance in mental resilience (*R*^2^ = 0.37, adjusted *R*^2^ = 0.35). Sustainable sport nutrition practices (β = 0.32, *p* < 0.001), recovery quality (β = 0.29, *p* < 0.001), and sustainability awareness (β = 0.21, *p* = 0.003) were positively associated with mental resilience after adjustment for the remaining model variables. The second regression model examined variables statistically associated with perceived athletic performance.

**Figure 6:**
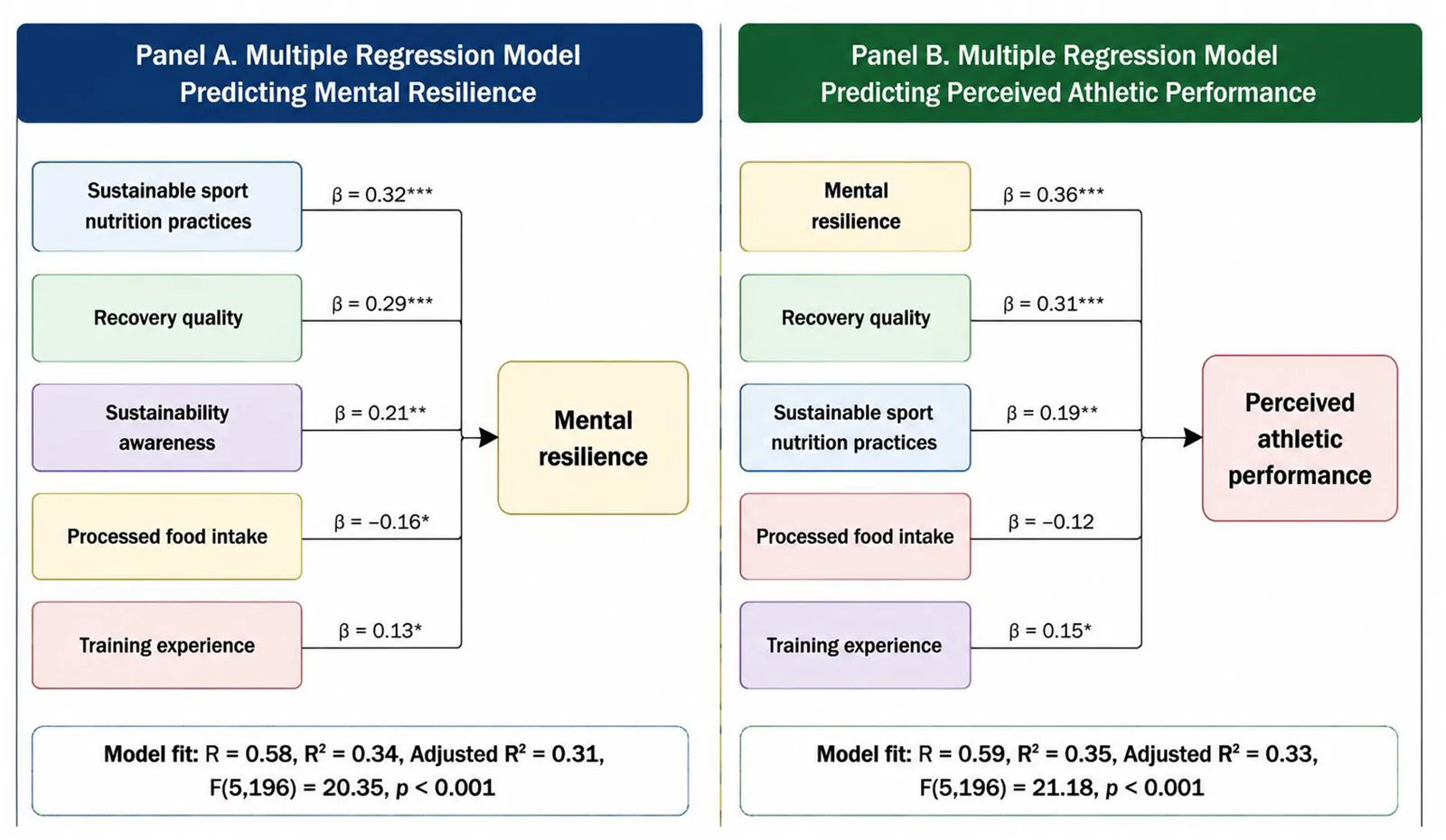
Independent multiple regression association models. Panel **(A)** presents predictors statistically associated with mental resilience, and Panel **(B)** presents predictors statistically associated with perceived athletic performance. The symbols represent statistical significance: ^*^p < 0.05, ^*^^*^p < 0.01, and ^*^^*^^*^p < 0.001.

This model was also statistically significant, *F*(5,196) = 18.91, p < 0.001, and accounted for 33% of the variance in perceived athletic performance (*R*^2^ = 0.33, adjusted *R*^2^ = 0.31). Mental resilience showed the strongest positive association with perceived performance (β = 0.36, *p* < 0.001), followed by recovery quality (β = 0.31, p < 0.001) and sustainable sport nutrition practices (β = 0.19, *p* = 0.008) (23). These results suggest that sustainable sport nutrition practices were statistically associated with perceived athletic performance, while recovery quality and mental resilience showed additional independent associations within the regression model. However, because the study design was cross-sectional, these findings should not be interpreted as evidence of causal, temporal, or mediation pathways (24). Longitudinal or intervention-based studies are required to test whether sustainable sport nutrition practices lead to changes in recovery, resilience, or performance over time as seen in Table 3.

**Table 3:** Multiple regression association models for mental resilience and perceived athletic performance.

Model/outcome	Predictor	*B*	SE	β	*t*	*p*-value	Tolerance/VIF
Model A: Mental resilience	Sustainable sport nutrition practices	0.27	0.052	0.32	5.18	< 0.001	0.68/1.48
Model A: Mental resilience	Recovery quality	0.30	0.064	0.29	4.69	< 0.001	0.73/1.37
Model A: Mental resilience	Sustainability awareness	0.20	0.066	0.21	3.02	0.003	0.70/1.42
Model A: Mental resilience	Processed food intake	−0.12	0.058	−0.13	−2.05	0.042	0.86/1.16
Model A: Mental resilience	Training experience	0.02	0.016	0.06	1.00	0.319	0.95/1.05
Model B: Perceived athletic performance	Mental resilience	0.34	0.065	0.36	5.19	< 0.001	0.62/1.62
Model B: Perceived athletic performance	Recovery quality	0.29	0.066	0.31	4.45	< 0.001	0.67/1.49
Model B: Perceived athletic performance	Sustainable sport nutrition practices	0.15	0.056	0.19	2.67	0.008	0.72/1.39
Model B: Perceived athletic performance	Processed food intake	−0.06	0.056	−0.07	−1.04	0.299	0.88/1.14
Model B: Perceived athletic performance	Training experience	0.02	0.016	0.08	1.20	0.232	0.94/1.06

Model A outcome variable: mental resilience. Model B outcome variable: perceived athletic performance. *B*, unstandardized regression coefficient; SE, standard error; β, standardized regression coefficient; VIF, variance inflation factor. Values indicate no serious multicollinearity, as all VIF values were below 5.0 and all tolerance values were above 0.20.

### 3.7 Qualitative findings and mixed-methods integration

The qualitative findings were integrated with the quantitative results using a joint display approach. Four major themes were identified: sustainable diet as mental preparation, recovery confidence, emotional stability and focus, and practical barriers to sustainable eating (25). These themes helped explain why sustainable sport nutrition practices were statistically associated with mental resilience, recovery quality, and perceived performance (26). Athletes described structured meals, hydration routines, and reduced dependence on highly processed foods as part of a broader preparation routine. These experiences aligned with the quantitative association between sustainable sport nutrition practices and mental resilience (27). Similarly, athletes who described better recovery confidence often emphasized regular meals, sufficient fluids, and feeling less sluggish during training, which helped contextualize the positive association between sustainable sport nutrition practices and recovery quality (28). The qualitative findings also added nuance to the negative association between processed food intake and resilience. As shown in Table 4, rather than indicating that processed foods directly reduced resilience, interviews suggested that processed food reliance often reflected practical constraints such as time pressure, food availability, academic or work schedules, travel, and cost (29). Thus, the qualitative results helped interpret the quantitative associations within the everyday realities of competitive athletes.

**Table 4:** Joint display integrating quantitative findings with qualitative themes.

Quantitative finding	Qualitative theme	Representative quote	Integrated interpretation
Sustainable sport nutrition practices were associated with mental resilience: *r* = 0.47, *p* < 0.001.	Sustainable diet as mental preparation	“When I plan my meals properly and avoid random junk food, I feel more disciplined and mentally ready for training. It gives me confidence that I have prepared myself well.”	Athletes interpreted structured eating as a self-regulation routine linked with discipline, focus, and confidence.
Sustainable sport nutrition practices were associated with recovery quality: *r* = 0.43, *p* < 0.001.	Recovery confidence	“After balanced meals and enough fluids, I feel less heavy and recover better for the next session. I feel my body is ready again instead of feeling tired all day.”	Quantitative recovery associations were supported by athlete descriptions of feeling more physically restored and ready for the next session.
Mental resilience was positively correlated with perceived athletic performance (*r* = 0.52, *p* < 0.001), as shown in the correlation matrix (Figure 5). This relationship represents the association between psychological resilience and performance perception and is distinct from the correlation between sustainable sport nutrition practices and perceived performance (*r* = 0.39, *p* < 0.001).	Emotional stability and focus	“When I stay calm and focused, I usually feel that I perform better. Even if training is hard, confidence helps me keep control and continue.”	Athletes described confidence, calmness, and focus as central to how they judged their performance readiness.
Sustainability awareness was associated with sustainable sport nutrition practices: *r* = 0.51, *p* < 0.001.	Value-based food choices	“I know food choices affect my body and also the environment, so I try to choose better foods when I can. But sometimes time, cost, and availability make it difficult.”	Awareness appeared to support more intentional food choices, but did not guarantee consistent practice.
Processed food intake was negatively associated with mental resilience: *r* = −0.31, *p* < 0.001.	Practical barriers and food access	“Sometimes I eat processed food because it is fast and cheap, not because I think it is better. When my schedule is busy, it becomes difficult to find fresh meals.”	Interviews suggested that processed food intake may reflect time pressure, cost, travel, and limited food access rather than diet preference alone.

## 4 Discussion

The present study examined cross-sectional associations between sustainable sport nutrition practices, mental resilience, recovery quality, and perceived athletic performance among competitive athletes. Because this study used a cross-sectional design, the observed regression associations should not be interpreted as evidence that sustainable sport nutrition practices caused improvements in resilience, recovery, or perceived performance (30). Rather, the findings identify statistically significant relationships among these variables within the present athlete sample. The conceptual and regression figures are therefore intended to summarize theoretically informed associations, not temporal, causal, or mediation mechanisms. A central finding of this study is the positive association between sustainable sport nutrition practices and mental resilience. Athletes who reported more consistent sustainable sport nutrition behaviors also reported stronger focus, emotional control, confidence, and stress tolerance. This association may be partly explained through self-regulation: athletes who plan meals, hydrate consistently, reduce reliance on highly processed foods, and make deliberate food choices may also have more structured routines that support psychological preparedness (31). It may also reflect value-based alignment, where athletes experience dietary choices as meaningful, responsible, and connected with long-term health and identity.

A key issue in applying sustainable nutrition to athletes is the need to protect energy availability and protein adequacy. Sustainable sport nutrition should not be reduced to eating less animal protein or lowering total food intake. Competitive athletes require sufficient energy, carbohydrate availability, protein quantity and quality, essential amino acids, leucine-rich foods, hydration, and micronutrients to support training adaptation and recovery (32). Therefore, a plant-forward dietary pattern may be appropriate only when it is carefully planned to meet these demands. The present Sustainable Sport Nutrition Practice Score included meal planning and protein adequacy domains, but it did not directly quantify energy intake, protein intake per kilogram of body mass, leucine intake, or biomarkers of low energy availability. The findings should therefore be interpreted as associations between self-reported sustainable sport nutrition behaviors and psychological/performance-related perceptions, not as proof that all sustainable diets meet athlete physiological requirements. The association between sustainable sport nutrition practices and recovery quality provides an important explanation for the psychological benefits reported by athletes. Recovery is not only a physiological process; it is also a psychological experience. Athletes who believe they are recovering well may enter training and competition with greater confidence and lower anxiety. In this study, recovery quality was associated with both mental resilience and perceived performance, suggesting that recovery may be a key related construct linking dietary behavior with psychological readiness.

The regression association model showed that mental resilience was the strongest variable associated with perceived athletic performance. This finding is meaningful because it highlights the psychological appraisal component of performance perception. Sustainable sport nutrition practices were associated with perceived performance, but their broader relevance appeared to occur in relation to resilience and recovery. This suggests that dietary behavior may be embedded within a wider performance system involving emotional regulation, motivation, confidence, and training discipline. Sustainability awareness was also associated with sustainable sport nutrition practices and mental resilience (33). This indicates that athletes with higher awareness of food quality, environmental impact, and responsible consumption were more likely to adopt sustainable sport nutrition behaviors. From a psychological perspective, this may reflect value-based motivation. When athletes believe that their dietary choices are beneficial not only for performance but also for long-term health and environmental responsibility, they may experience stronger internal motivation and self-discipline.

The negative association between processed food intake and mental resilience should not be interpreted simplistically. Highly processed foods are often convenient, affordable, energy-dense, and accessible, especially for athletes managing training, study, work, travel, or limited food environments. Therefore, higher processed food intake may partly reflect socioeconomic status, food insecurity, time constraints, training stress, or lack of access to fresh foods. These contextual factors were not fully measured in the present study and may confound the observed association. Future research should include socioeconomic indicators such as household income, financial food allowance, food security status, and access to affordable nutritious foods as covariates. These factors may influence processed food consumption patterns and help determine whether observed associations reflect dietary behavior itself or broader environmental constraints.

The qualitative findings strengthened the quantitative results by showing how athletes interpret diet as part of their training identity. Athletes described balanced eating and structured hydration as preparation routines that helped them feel focused, calm, and confident before training or competition. These responses suggest that athletes do not experience nutrition only as a biological requirement; they also experience it as a psychological signal of discipline and readiness. However, the qualitative findings also showed that awareness alone is not sufficient for consistent sustainable sport nutrition behavior. Practical barriers, including cost, limited food availability, lack of time, taste preference, and uncertainty about protein adequacy, limited consistent adoption.

### 4.1 Practical implications for coaches, dietitians, and sport organizations

Sustainable sport nutrition should be implemented in a practical and performance-protective way. Coaches and dietitians should avoid presenting sustainable eating as a restrictive or low-energy dietary pattern. For competitive athletes, sustainable nutrition must first meet total energy availability, carbohydrate needs, protein requirements, hydration demands, and recovery goals. Therefore, a useful approach is to promote plant-forward but not necessarily fully plant-based eating, where athletes increase whole grains, legumes, fruits, vegetables, nuts, seeds, and minimally processed foods while still including dairy, eggs, fish, poultry, or other culturally acceptable protein sources when needed.

Concrete strategies may include building high-energy meals around rice, pasta, oats, potatoes, whole-grain bread, beans, lentils, tofu, eggs, yogurt, milk, lean meat, fish, nuts, nut butter, olive oil, and smoothies. For example, an athlete's recovery meal could include rice or pasta, lentils or chicken, vegetables, yogurt, fruit, and fluids. A sustainable recovery snack could include Greek yogurt with fruit and oats, a peanut-butter banana sandwich, milk with cereal, or a smoothie containing milk or soy milk, banana, oats, and nut butter. These examples show that sustainable dietary choices can support both environmental responsibility and sport-specific fueling.

Dietitians should also provide clear protein guidance. Athletes may worry that sustainable diets reduce protein quality or quantity. This concern can be addressed by teaching athletes to combine diverse protein sources across the day, such as legumes with grains, soy products, dairy, eggs, fish, poultry, meat, nuts, and seeds. Rather than removing animal foods completely, athletes can be encouraged to reduce reliance on highly processed foods and increase the proportion of nutrient-dense, lower-waste, and minimally processed foods (34). At the team or institutional level, practical changes may include offering balanced cafeteria meals with both plant-based and mixed-protein options, providing portable recovery snacks after training, ensuring water access at training sites, reducing food waste through pre-ordering systems, and aligning menus with training schedules. Coaches can support this process by treating nutrition routines as part of preparation and recovery, not as an optional lifestyle message. In this way, sustainable nutrition can be framed as a realistic performance-supportive behavior that protects energy intake, recovery quality, and psychological readiness.

### 4.2 Limitations and future research

This study has several limitations. First, the cross-sectional design does not allow causal or temporal conclusions. Although sustainable sport nutrition practices were associated with mental resilience, recovery quality, and perceived performance, the direction of these relationships cannot be established. It is also possible that athletes with stronger resilience or better perceived performance are more likely to maintain structured dietary routines. Longitudinal, intervention-based, or experimental studies are needed to test causal mechanisms. Second, dietary practices, recovery quality, mental resilience, and perceived athletic performance were self-reported, which may introduce recall bias, social desirability bias, and common-method bias. Because the variables were measured using self-report questionnaires at the same time point, athletes with higher resilience, confidence, or positive affect may have rated their diet, recovery, and performance more favorably regardless of objective performance. Therefore, the association between mental resilience and perceived performance may partly reflect shared psychological appraisal rather than actual performance differences.

Third, the study used perceived athletic performance rather than objective performance indicators such as race time, strength output, match statistics, coach ratings, physiological recovery markers, or training-load data. Future studies should include objective dietary records, biomarkers, sleep tracking, recovery monitoring, direct performance tests, and longitudinal performance records. Fourth, the sample mainly represented university and club/regional competitive athletes, with a smaller national-level subgroup. Therefore, the findings should not be generalized directly to elite, professional, or Olympic-level athletes, who may have different training loads, nutrition support systems, travel demands, and performance pressures. Fifth, Therefore, the associations observed in this study should be interpreted as relationships involving the operationalized behavioral index used here rather than a fully validated measure of sustainable sport nutrition behavior. The scale should be further tested using factor analysis, test–retest reliability, convergent validity, and criterion validity. The scale also did not directly measure total energy intake, protein intake per kilogram of body mass, leucine intake, amino acid distribution, or low energy availability risk.

Sixth, the hydration item should be interpreted cautiously. It measured hydration consistency as a sport nutrition behavior, not environmentally sustainable hydration practices such as reusable bottle use or reduced single-use plastic consumption. Future studies should separate ecological sustainability items from general sport nutrition behaviors. Future research should also compare team and individual sports, male and female athletes, and different competition levels to identify whether sustainable nutrition–resilience associations differ across athlete groups.

## 5 Conclusion

This study investigated sustainable sport nutrition behaviors and mental resilience from a psychological perspective on athletic performance. The findings showed that sustainable sport nutrition practices were positively associated with mental resilience, recovery quality, and perceived athletic performance. Regression analysis indicated that sustainable sport nutrition practices, recovery quality, and sustainability awareness were statistically associated with mental resilience, while mental resilience and recovery quality were associated with perceived athletic performance. Qualitative findings further suggested that athletes viewed balanced and sustainable eating as part of mental preparation, emotional control, recovery confidence, and training discipline. However, barriers such as cost, access, time limitations, taste preference, and protein concerns limited consistent adoption. These findings suggest that sustainable sport nutrition should be promoted as practical, performance-compatible, energy-adequate, and protein-sufficient rather than only as an environmental behavior. Because the study was cross-sectional and relied on self-report measures, the findings should be interpreted as associations rather than causal evidence. Future studies should use longitudinal designs, objective dietary records, validated instruments, and direct performance measures to confirm these findings over time.

## Data Availability

The raw data supporting the conclusions of this article will be made available by the authors, without undue reservation.
