# Supplementary material for: Sustainable sport nutrition behaviors, mental resilience, and perceived athletic performance in competitive athletes
Source: Front Nutr. 2026 Oct 6;13:1935463. doi: 10.3389/fnut.2026.1935463 (PMC13680215; doi:10.3389/fnut.2026.1935463)
Supplement: Supplementary file 1 [file Supplementary_file_1.docx]

# Supplementary materials

Supplementary Table S1. Items of the Sustainable Sport Nutrition Practice Score.

| Item no. | Item content | Response format |
| --- | --- | --- |
| 1 | I include plant-forward meals such as vegetables, fruits, legumes, whole grains, nuts, or seeds in my regular diet. | 1 = never to 5 = always |
| 2 | I choose whole or minimally processed foods instead of highly processed foods when possible. | 1 = never to 5 = always |
| 3 | I maintain regular hydration before, during, and after training. | 1 = never to 5 = always |
| 4 | I plan meals or snacks to support training and recovery. | 1 = never to 5 = always |
| 5 | I include adequate protein from diverse sources such as legumes, dairy, eggs, soy, fish, poultry, meat, nuts, or seeds. | 1 = never to 5 = always |
| 6 | I choose local or seasonal foods when available and practical. | 1 = never to 5 = always |
| 7 | I try to reduce food waste by planning portions or reusing leftovers safely. | 1 = never to 5 = always |
| 8 | I consider both performance needs and environmental responsibility when making food choices. | 1 = never to 5 = always |

Supplementary Table S2. Qualitative coding tree for athlete interviews.

| Major theme | Sub-theme | Meaning in the data | Representative quote |
| --- | --- | --- | --- |
| Sustainable diet as mental preparation | Meal planning as self-regulation | Athletes described planned meals as helping them feel organized, disciplined, and ready for training. | “When I prepare my meals before training days, I feel more organized. It makes me feel that I am controlling my routine instead of leaving everything to chance.” |
| Sustainable diet as mental preparation | Food choice and confidence | Athletes linked purposeful food choices with confidence before training or competition. | “When I eat the right foods before training or competition, I feel more confident because I know I have prepared my body properly.” |
| Recovery confidence | Feeling physically restored | Athletes associated balanced meals, hydration, and lower processed food intake with better perceived recovery. | “After I eat balanced meals and drink enough water, I feel my body recovers better. I wake up feeling more ready for the next session.” |
| Recovery confidence | Reduced heaviness or sluggishness | Some athletes reported feeling lighter or less sluggish after more balanced meals. | “When I reduce fast food and eat more balanced meals, I feel less heavy during training. My body feels lighter and I do not feel tired so quickly.” |
| Emotional stability and focus | Stable energy and mood | Athletes described regular meals and hydration as helping them feel calmer and more focused. | “If I skip meals or eat badly, my mood changes and I lose focus. When I eat regularly and stay hydrated, I feel calmer and more stable.” |
| Emotional stability and focus | Sense of control | Athletes interpreted nutrition routines as something they could control during stressful training periods. | “Training pressure is not always under my control, but my food routine is something I can manage. That gives me a sense of control.” |
| Practical barriers | Time, cost, and access | Athletes reported that busy schedules, limited food access, and cost affected sustainable food choices. | “Sometimes I want to eat healthier and more sustainable food, but after training I do not have enough time or money, so I choose whatever is quick and available.” |
| Practical barriers | Protein concerns | Some athletes were unsure whether plant-forward meals could meet sport protein needs. | “I am interested in eating more plant-based foods, but I worry whether I can get enough protein for recovery and strength.” |
